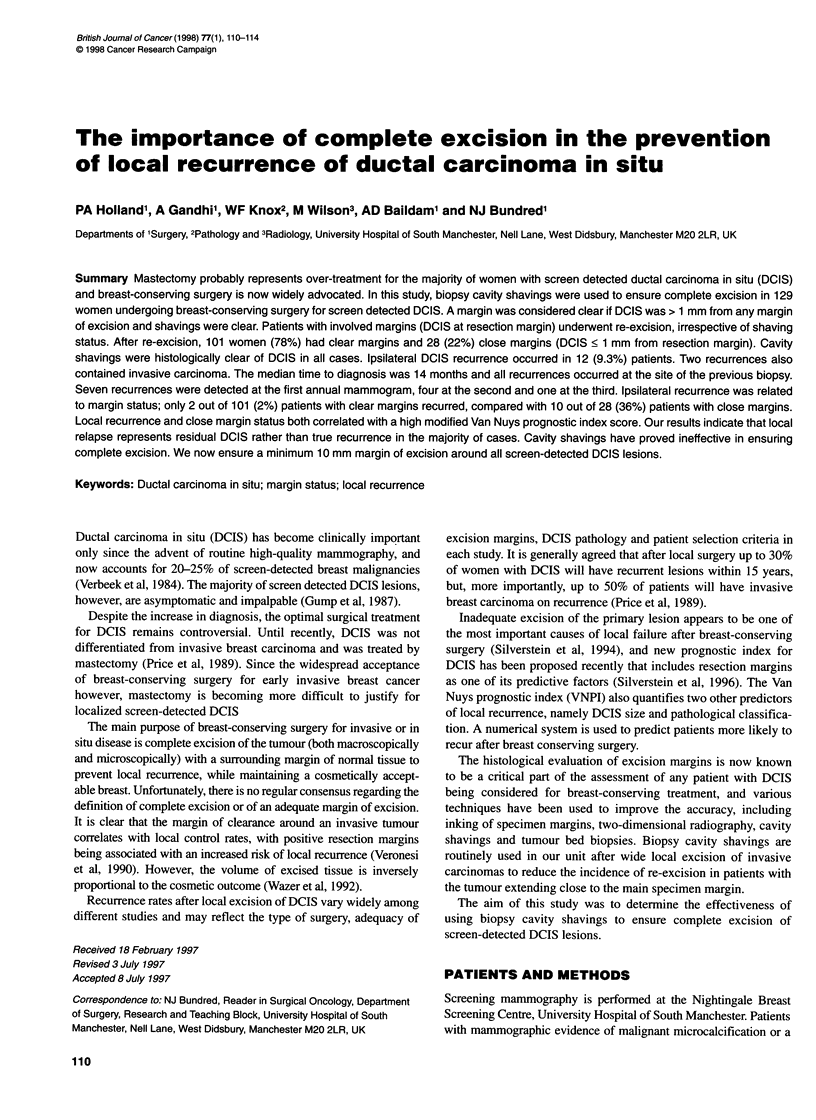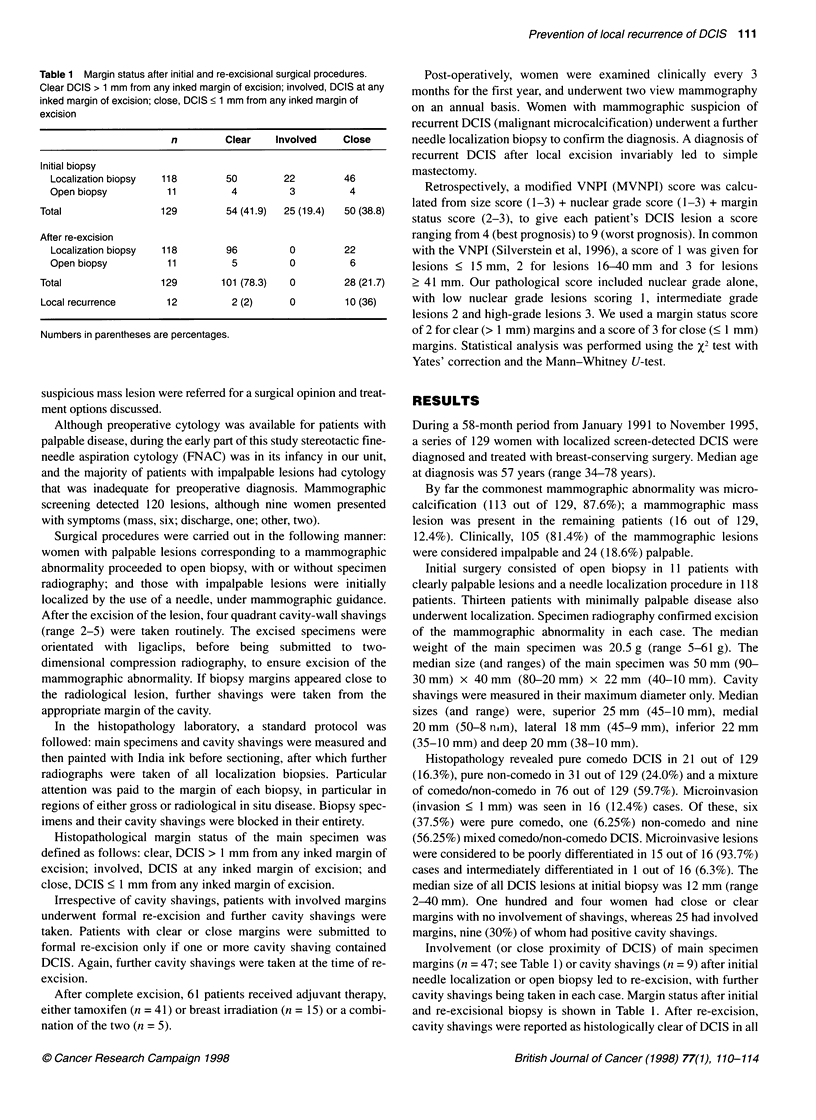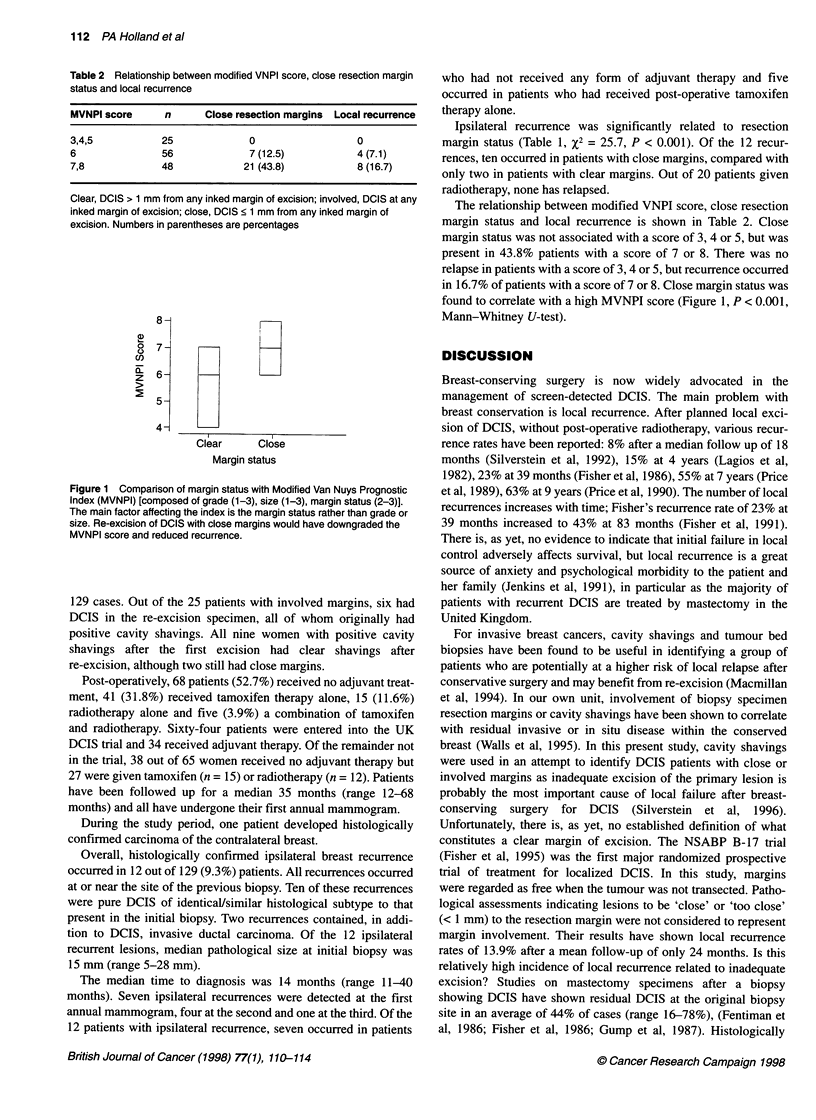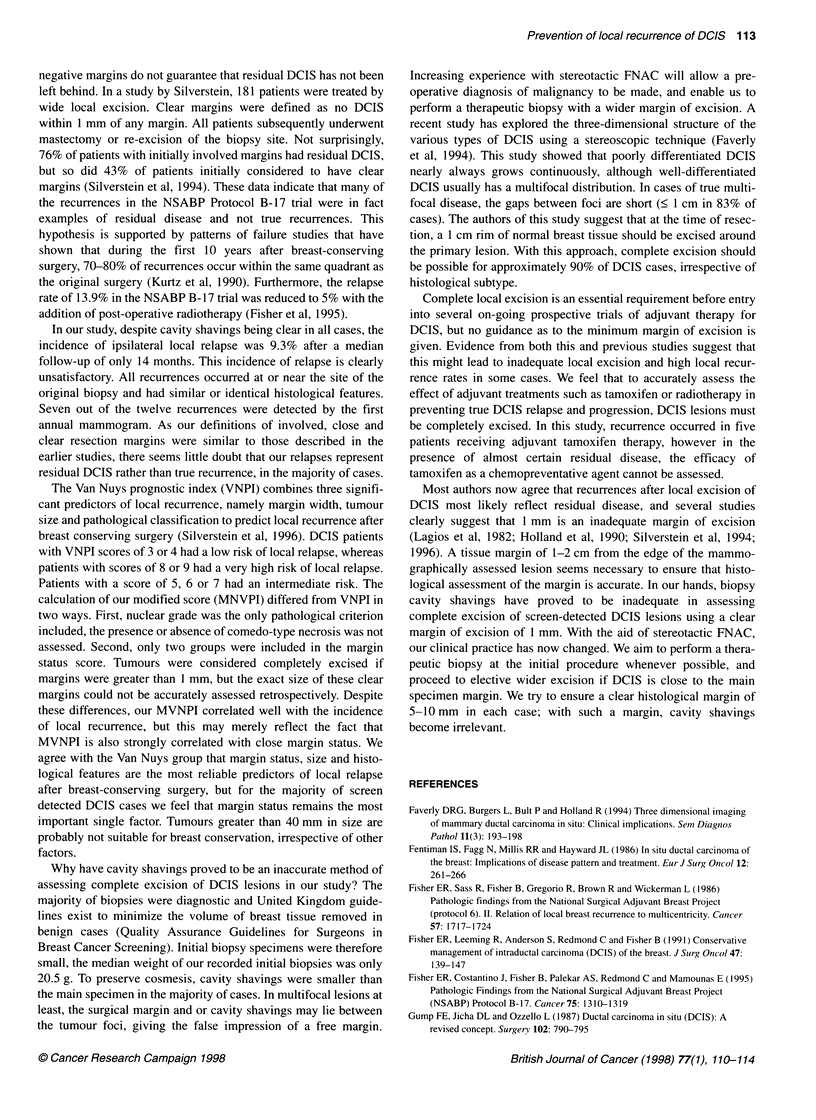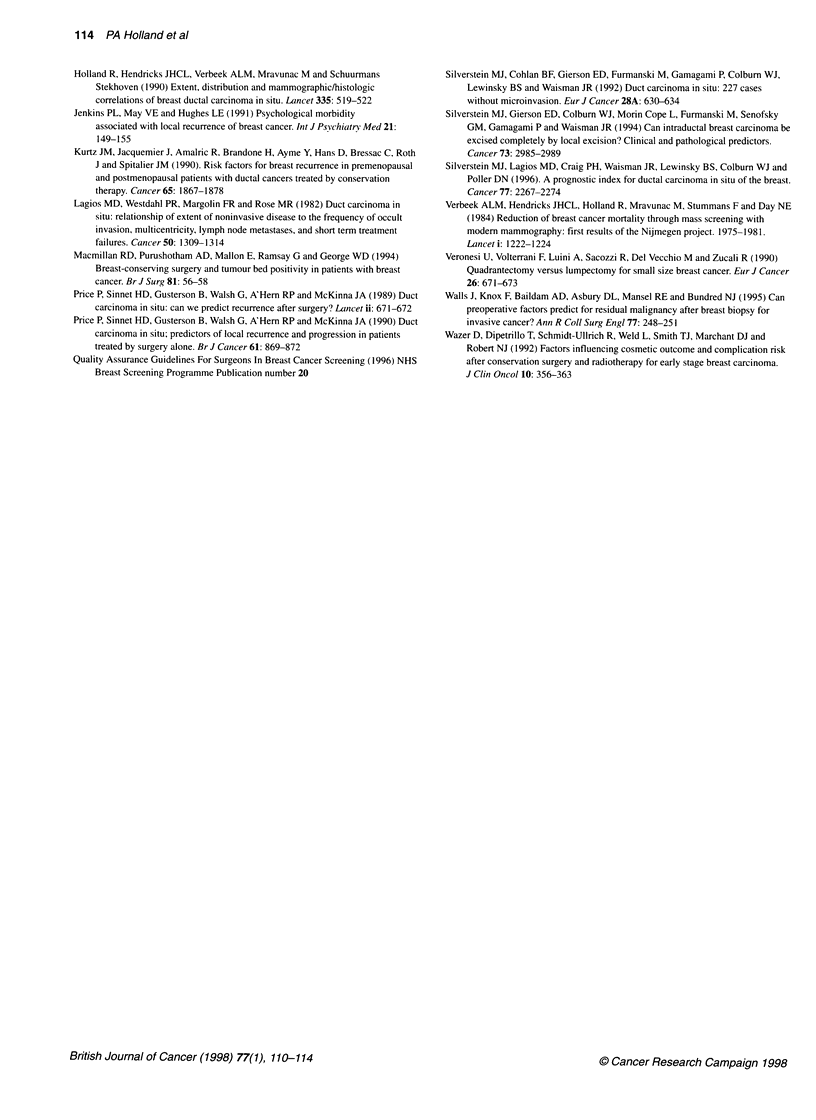# The importance of complete excision in the prevention of local recurrence of ductal carcinoma in situ.

**DOI:** 10.1038/bjc.1998.17

**Published:** 1998

**Authors:** P. A. Holland, A. Gandhi, W. F. Knox, M. Wilson, A. D. Baildam, N. J. Bundred

**Affiliations:** Department of Surgery, University Hospital of South Manchester, West Didsbury, UK.

## Abstract

Mastectomy probably represents over-treatment for the majority of women with screen detected ductal carcinoma in situ (DCIS) and breast-conserving surgery is now widely advocated. In this study, biopsy cavity shavings were used to ensure complete excision in 129 women undergoing breast-conserving surgery for screen detected DCIS. A margin was considered clear if DCIS was > 1 mm from any margin of excision and shavings were clear. Patients with involved margins (DCIS at resection margin) underwent re-excision, irrespective of shaving status. After re-excision, 101 women (78%) had clear margins and 28 (22%) close margins (DCIS < or = 1 mm from resection margin). Cavity shavings were histologically clear of DCIS in all cases. Ipsilateral DCIS recurrence occurred in 12 (9.3%) patients. Two recurrences also contained invasive carcinoma. The median time to diagnosis was 14 months and all recurrences occurred at the site of the previous biopsy. Seven recurrences were detected at the first annual mammogram, four at the second and one at the third. Ipsilateral recurrence was related to margin status; only 2 out of 101 (2%) patients with clear margins recurred, compared with 10 out of 28 (36%) patients with close margins. Local recurrence and close margin status both correlated with a high modified Van Nuys prognostic index score. Our results indicate that local relapse represents residual DCIS rather than true recurrence in the majority of cases. Cavity shavings have proved ineffective in ensuring complete excision. We now ensure a minimum 10 mm margin of excision around all screen-detected DCIS lesions.


*British Journal of Cancer* (1998) **77**(1), 110–114
© 1998 Cancer Research Campaign

### The importance of complete excision in the prevention of local recurrence of ductal carcinoma in situ

**PA Holland[1], A Gandhi[1], WF Knox[2], M Wilson[3], AD Baildam[1] and NJ Bundred[1]**

Departments of [1]Surgery, [2]Pathology and [3]Radiology, University Hospital of South Manchester, Nell Lane, West Didsbury, Manchester M20 2LR, UK

**Summary** Mastectomy probably represents over-treatment for the majority of women with screen detected ductal carcinoma in situ (DCIS) and breast-conserving surgery is now widely advocated. In this study, biopsy cavity shavings were used to ensure complete excision in 129 women undergoing breast-conserving surgery for screen detected DCIS. A margin was considered clear if DCIS was > 1 mm from any margin of excision and shavings were clear. Patients with involved margins (DCIS at resection margin) underwent re-excision, irrespective of shaving status. After re-excision, 101 women (78%) had clear margins and 28 (22%) close margins (DCIS ≤ 1 mm from resection margin). Cavity shavings were histologically clear of DCIS in all cases. Ipsilateral DCIS recurrence occurred in 12 (9.3%) patients. Two recurrences also contained invasive carcinoma. The median time to diagnosis was 14 months and all recurrences occurred at the site of the previous biopsy. Seven recurrences were detected at the first annual mammogram, four at the second and one at the third. Ipsilateral recurrence was related to margin status; only 2 out of 101 (2%) patients with clear margins recurred, compared with 10 out of 28 (36%) patients with close margins. Local recurrence and close margin status both correlated with a high modified Van Nuys prognostic index score. Our results indicate that local relapse represents residual DCIS rather than true recurrence in the majority of cases. Cavity shavings have proved ineffective in ensuring complete excision. We now ensure a minimum 10 mm margin of excision around all screen-detected DCIS lesions.

**Keywords:** Ductal carcinoma in situ; margin status; local recurrence

Ductal carcinoma in situ (DCIS) has become clinically important only since the advent of routine high-quality mammography, and now accounts for 20–25% of screen-detected breast malignancies (Verbeek et al, 1984). The majority of screen detected DCIS lesions, however, are asymptomatic and impalpable (Gump et al, 1987).

Despite the increase in diagnosis, the optimal surgical treatment for DCIS remains controversial. Until recently, DCIS was not differentiated from invasive breast carcinoma and was treated by mastectomy (Price et al, 1989). Since the widespread acceptance of breast-conserving surgery for early invasive breast cancer however, mastectomy is becoming more difficult to justify for localized screen-detected DCIS

The main purpose of breast-conserving surgery for invasive or in situ disease is complete excision of the tumour (both macroscopically and microscopically) with a surrounding margin of normal tissue to prevent local recurrence, while maintaining a cosmetically acceptable breast. Unfortunately, there is no regular consensus regarding the definition of complete excision or of an adequate margin of excision. It is clear that the margin of clearance around an invasive tumour correlates with local control rates, with positive resection margins being associated with an increased risk of local recurrence (Veronesi et al, 1990). However, the volume of excised tissue is inversely proportional to the cosmetic outcome (Wazer et al, 1992).

Recurrence rates after local excision of DCIS vary widely among different studies and may reflect the type of surgery, adequacy of excision margins, DCIS pathology and patient selection criteria in each study. It is generally agreed that after local surgery up to 30% of women with DCIS will have recurrent lesions within 15 years, but, more importantly, up to 50% of patients will have invasive breast carcinoma on recurrence (Price et al, 1989).

Inadequate excision of the primary lesion appears to be one of the most important causes of local failure after breast-conserving surgery (Silverstein et al, 1994), and new prognostic index for DCIS has been proposed recently that includes resection margins as one of its predictive factors (Silverstein et al, 1996). The Van Nuys prognostic index (VNPI) also quantifies two other predictors of local recurrence, namely DCIS size and pathological classification. A numerical system is used to predict patients more likely to recur after breast conserving surgery.

The histological evaluation of excision margins is now known to be a critical part of the assessment of any patient with DCIS being considered for breast-conserving treatment, and various techniques have been used to improve the accuracy, including inking of specimen margins, two-dimensional radiography, cavity shavings and tumour bed biopsies. Biopsy cavity shavings are routinely used in our unit after wide local excision of invasive carcinomas to reduce the incidence of re-excision in patients with the tumour extending close to the main specimen margin.

The aim of this study was to determine the effectiveness of using biopsy cavity shavings to ensure complete excision of screen-detected DCIS lesions.

*Received 18 February 1997*
*Revised 3 July 1997*
*Accepted 8 July 1997*

*Correspondence to:* NJ Bundred, Reader in Surgical Oncology, Department of Surgery, Research and Teaching Block, University Hospital of South Manchester, Nell Lane, West Didsbury, Manchester M20 2LR, UK

### PATIENTS AND METHODS

Screening mammography is performed at the Nightingale Breast Screening Centre, University Hospital of South Manchester. Patients with mammographic evidence of malignant microcalcification or a

**Table 1** Margin status after initial and re-excisional surgical procedures. Clear DCIS > 1 mm from any inked margin of excision; involved, DCIS at any inked margin of excision; close, DCIS ≤ 1 mm from any inked margin of excision

| | *n* | Clear | Involved | Close |
|---|---|---|---|---|
| Initial biopsy | | | | |
| Localization biopsy | 118 | 50 | 22 | 46 |
| Open biopsy | 11 | 4 | 3 | 4 |
| Total | 129 | 54 (41.9) | 25 (19.4) | 50 (38.8) |
| After re-excision | | | | |
| Localization biopsy | 118 | 96 | 0 | 22 |
| Open biopsy | 11 | 5 | 0 | 6 |
| Total | 129 | 101 (78.3) | 0 | 28 (21.7) |
| Local recurrence | 12 | 2 (2) | 0 | 10 (36) |

Numbers in parentheses are percentages.

suspicious mass lesion were referred for a surgical opinion and treatment options discussed.

Although preoperative cytology was available for patients with palpable disease, during the early part of this study stereotactic fine-needle aspiration cytology (FNAC) was in its infancy in our unit, and the majority of patients with impalpable lesions had cytology that was inadequate for preoperative diagnosis. Mammographic screening detected 120 lesions, although nine women presented with symptoms (mass, six; discharge, one; other, two).

Surgical procedures were carried out in the following manner: women with palpable lesions corresponding to a mammographic abnormality proceeded to open biopsy, with or without specimen radiography; and those with impalpable lesions were initially localized by the use of a needle, under mammographic guidance. After the excision of the lesion, four quadrant cavity-wall shavings (range 2–5) were taken routinely. The excised specimens were orientated with ligaclips, before being submitted to two-dimensional compression radiography, to ensure excision of the mammographic abnormality. If biopsy margins appeared close to the radiological lesion, further shavings were taken from the appropriate margin of the cavity.

In the histopathology laboratory, a standard protocol was followed: main specimens and cavity shavings were measured and then painted with India ink before sectioning, after which further radiographs were taken of all localization biopsies. Particular attention was paid to the margin of each biopsy, in particular in regions of either gross or radiological in situ disease. Biopsy specimens and their cavity shavings were blocked in their entirety.

Histopathological margin status of the main specimen was defined as follows: clear, DCIS > 1 mm from any inked margin of excision; involved, DCIS at any inked margin of excision; and close, DCIS ≤ 1 mm from any inked margin of excision.

Irrespective of cavity shavings, patients with involved margins underwent formal re-excision and further cavity shavings were taken. Patients with clear or close margins were submitted to formal re-excision only if one or more cavity shaving contained DCIS. Again, further cavity shavings were taken at the time of re-excision.

After complete excision, 61 patients received adjuvant therapy, either tamoxifen ($n = 41$) or breast irradiation ($n = 15$) or a combination of the two ($n = 5$).

Post-operatively, women were examined clinically every 3 months for the first year, and underwent two view mammography on an annual basis. Women with mammographic suspicion of recurrent DCIS (malignant microcalcification) underwent a further needle localization biopsy to confirm the diagnosis. A diagnosis of recurrent DCIS after local excision invariably led to simple mastectomy.

Retrospectively, a modified VNPI (MVNPI) score was calculated from size score (1–3) + nuclear grade score (1–3) + margin status score (2–3), to give each patient's DCIS lesion a score ranging from 4 (best prognosis) to 9 (worst prognosis). In common with the VNPI (Silverstein et al, 1996), a score of 1 was given for lesions ≤ 15 mm, 2 for lesions 16–40 mm and 3 for lesions ≥ 41 mm. Our pathological score included nuclear grade alone, with low nuclear grade lesions scoring 1, intermediate grade lesions 2 and high-grade lesions 3. We used a margin status score of 2 for clear (> 1 mm) margins and a score of 3 for close (≤ 1 mm) margins. Statistical analysis was performed using the $\chi^2$ test with Yates' correction and the Mann–Whitney *U*-test.

### RESULTS

During a 58-month period from January 1991 to November 1995, a series of 129 women with localized screen-detected DCIS were diagnosed and treated with breast-conserving surgery. Median age at diagnosis was 57 years (range 34–78 years).

By far the commonest mammographic abnormality was microcalcification (113 out of 129, 87.6%); a mammographic mass lesion was present in the remaining patients (16 out of 129, 12.4%). Clinically, 105 (81.4%) of the mammographic lesions were considered impalpable and 24 (18.6%) palpable.

Initial surgery consisted of open biopsy in 11 patients with clearly palpable lesions and a needle localization procedure in 118 patients. Thirteen patients with minimally palpable disease also underwent localization. Specimen radiography confirmed excision of the mammographic abnormality in each case. The median weight of the main specimen was 20.5 g (range 5–61 g). The median size (and ranges) of the main specimen was 50 mm (90–30 mm) × 40 mm (80–20 mm) × 22 mm (40–10 mm). Cavity shavings were measured in their maximum diameter only. Median sizes (and range) were, superior 25 mm (45–10 mm), medial 20 mm (50–8 mm), lateral 18 mm (45–9 mm), inferior 22 mm (35–10 mm) and deep 20 mm (38–10 mm).

Histopathology revealed pure comedo DCIS in 21 out of 129 (16.3%), pure non-comedo in 31 out of 129 (24.0%) and a mixture of comedo/non-comedo in 76 out of 129 (59.7%). Microinvasion (invasion ≤ 1 mm) was seen in 16 (12.4%) cases. Of these, six (37.5%) were pure comedo, one (6.25%) non-comedo and nine (56.25%) mixed comedo/non-comedo DCIS. Microinvasive lesions were considered to be poorly differentiated in 15 out of 16 (93.7%) cases and intermediately differentiated in 1 out of 16 (6.3%). The median size of all DCIS lesions at initial biopsy was 12 mm (range 2–40 mm). One hundred and four women had close or clear margins with no involvement of shavings, whereas 25 had involved margins, nine (30%) of whom had positive cavity shavings.

Involvement (or close proximity of DCIS) of main specimen margins ($n = 47$; see Table 1) or cavity shavings ($n = 9$) after initial needle localization or open biopsy led to re-excision, with further cavity shavings being taken in each case. Margin status after initial and re-excisional biopsy is shown in Table 1. After re-excision, cavity shavings were reported as histologically clear of DCIS in all

**Table 2** Relationship between modified VNPI score, close resection margin status and local recurrence

| MVNPI score | $n$ | Close resection margins | Local recurrence |
|---|---|---|---|
| 3,4,5 | 25 | 0 | 0 |
| 6 | 56 | 7 (12.5) | 4 (7.1) |
| 7,8 | 48 | 21 (43.8) | 8 (16.7) |

Clear, DCIS > 1 mm from any inked margin of excision; involved, DCIS at any inked margin of excision; close, DCIS ≤ 1 mm from any inked margin of excision. Numbers in parentheses are percentages

**Figure 1** Comparison of margin status with Modified Van Nuys Prognostic Index (MVNPI) [composed of grade (1–3), size (1–3), margin status (2–3)]. The main factor affecting the index is the margin status rather than grade or size. Re-excision of DCIS with close margins would have downgraded the MVNPI score and reduced recurrence.

129 cases. Out of the 25 patients with involved margins, six had DCIS in the re-excision specimen, all of whom originally had positive cavity shavings. All nine women with positive cavity shavings after the first excision had clear shavings after re-excision, although two still had close margins.

Post-operatively, 68 patients (52.7%) received no adjuvant treatment, 41 (31.8%) received tamoxifen therapy alone, 15 (11.6%) radiotherapy alone and five (3.9%) a combination of tamoxifen and radiotherapy. Sixty-four patients were entered into the UK DCIS trial and 34 received adjuvant therapy. Of the remainder not in the trial, 38 out of 65 women received no adjuvant therapy but 27 were given tamoxifen ($n$ = 15) or radiotherapy ($n$ = 12). Patients have been followed up for a median 35 months (range 12–68 months) and all have undergone their first annual mammogram.

During the study period, one patient developed histologically confirmed carcinoma of the contralateral breast.

Overall, histologically confirmed ipsilateral breast recurrence occurred in 12 out of 129 (9.3%) patients. All recurrences occurred at or near the site of the previous biopsy. Ten of these recurrences were pure DCIS of identical/similar histological subtype to that present in the initial biopsy. Two recurrences contained, in addition to DCIS, invasive ductal carcinoma. Of the 12 ipsilateral recurrent lesions, median pathological size at initial biopsy was 15 mm (range 5–28 mm).

The median time to diagnosis was 14 months (range 11–40 months). Seven ipsilateral recurrences were detected at the first annual mammogram, four at the second and one at the third. Of the 12 patients with ipsilateral recurrence, seven occurred in patients who had not received any form of adjuvant therapy and five occurred in patients who had received post-operative tamoxifen therapy alone.

Ipsilateral recurrence was significantly related to resection margin status (Table 1, $\chi^2 = 25.7$, $P < 0.001$). Of the 12 recurrences, ten occurred in patients with close margins, compared with only two in patients with clear margins. Out of 20 patients given radiotherapy, none has relapsed.

The relationship between modified VNPI score, close resection margin status and local recurrence is shown in Table 2. Close margin status was not associated with a score of 3, 4 or 5, but was present in 43.8% patients with a score of 7 or 8. There was no relapse in patients with a score of 3, 4 or 5, but recurrence occurred in 16.7% of patients with a score of 7 or 8. Close margin status was found to correlate with a high MVNPI score (Figure 1, $P < 0.001$, Mann–Whitney $U$-test).

### DISCUSSION

Breast-conserving surgery is now widely advocated in the management of screen-detected DCIS. The main problem with breast conservation is local recurrence. After planned local excision of DCIS, without post-operative radiotherapy, various recurrence rates have been reported: 8% after a median follow up of 18 months (Silverstein et al, 1992), 15% at 4 years (Lagios et al, 1982), 23% at 39 months (Fisher et al, 1986), 55% at 7 years (Price et al, 1989), 63% at 9 years (Price et al, 1990). The number of local recurrences increases with time; Fisher's recurrence rate of 23% at 39 months increased to 43% at 83 months (Fisher et al, 1991). There is, as yet, no evidence to indicate that initial failure in local control adversely affects survival, but local recurrence is a great source of anxiety and psychological morbidity to the patient and her family (Jenkins et al, 1991), in particular as the majority of patients with recurrent DCIS are treated by mastectomy in the United Kingdom.

For invasive breast cancers, cavity shavings and tumour bed biopsies have been found to be useful in identifying a group of patients who are potentially at a higher risk of local relapse after conservative surgery and may benefit from re-excision (Macmillan et al, 1994). In our own unit, involvement of biopsy specimen resection margins or cavity shavings have been shown to correlate with residual invasive or in situ disease within the conserved breast (Walls et al, 1995). In this present study, cavity shavings were used in an attempt to identify DCIS patients with close or involved margins as inadequate excision of the primary lesion is probably the most important cause of local failure after breast-conserving surgery for DCIS (Silverstein et al, 1996). Unfortunately, there is, as yet, no established definition of what constitutes a clear margin of excision. The NSABP B-17 trial (Fisher et al, 1995) was the first major randomized prospective trial of treatment for localized DCIS. In this study, margins were regarded as free when the tumour was not transected. Pathological assessments indicating lesions to be 'close' or 'too close' (< 1 mm) to the resection margin were not considered to represent margin involvement. Their results have shown local recurrence rates of 13.9% after a mean follow-up of only 24 months. Is this relatively high incidence of local recurrence related to inadequate excision? Studies on mastectomy specimens after a biopsy showing DCIS have shown residual DCIS at the original biopsy site in an average of 44% of cases (range 16–78%), (Fentiman et al, 1986; Fisher et al, 1986; Gump et al, 1987). Histologically

© Cancer Research Campaign 1998

negative margins do not guarantee that residual DCIS has not been left behind. In a study by Silverstein, 181 patients were treated by wide local excision. Clear margins were defined as no DCIS within 1 mm of any margin. All patients subsequently underwent mastectomy or re-excision of the biopsy site. Not surprisingly, 76% of patients with initially involved margins had residual DCIS, but so did 43% of patients initially considered to have clear margins (Silverstein et al, 1994). These data indicate that many of the recurrences in the NSABP Protocol B-17 trial were in fact examples of residual disease and not true recurrences. This hypothesis is supported by patterns of failure studies that have shown that during the first 10 years after breast-conserving surgery, 70–80% of recurrences occur within the same quadrant as the original surgery (Kurtz et al, 1990). Furthermore, the relapse rate of 13.9% in the NSABP B-17 trial was reduced to 5% with the addition of post-operative radiotherapy (Fisher et al, 1995).

In our study, despite cavity shavings being clear in all cases, the incidence of ipsilateral local relapse was 9.3% after a median follow-up of only 14 months. This incidence of relapse is clearly unsatisfactory. All recurrences occurred at or near the site of the original biopsy and had similar or identical histological features. Seven out of the twelve recurrences were detected by the first annual mammogram. As our definitions of involved, close and clear resection margins were similar to those described in the earlier studies, there seems little doubt that our relapses represent residual DCIS rather than true recurrence, in the majority of cases.

The Van Nuys prognostic index (VNPI) combines three significant predictors of local recurrence, namely margin width, tumour size and pathological classification to predict local recurrence after breast conserving surgery (Silverstein et al, 1996). DCIS patients with VNPI scores of 3 or 4 had a low risk of local relapse, whereas patients with scores of 8 or 9 had a very high risk of local relapse. Patients with a score of 5, 6 or 7 had an intermediate risk. The calculation of our modified score (MNVPI) differed from VNPI in two ways. First, nuclear grade was the only pathological criterion included, the presence or absence of comedo-type necrosis was not assessed. Second, only two groups were included in the margin status score. Tumours were considered completely excised if margins were greater than 1 mm, but the exact size of these clear margins could not be accurately assessed retrospectively. Despite these differences, our MVNPI correlated well with the incidence of local recurrence, but this may merely reflect the fact that MVNPI is also strongly correlated with close margin status. We agree with the Van Nuys group that margin status, size and histological features are the most reliable predictors of local relapse after breast-conserving surgery, but for the majority of screen detected DCIS cases we feel that margin status remains the most important single factor. Tumours greater than 40 mm in size are probably not suitable for breast conservation, irrespective of other factors.

Why have cavity shavings proved to be an inaccurate method of assessing complete excision of DCIS lesions in our study? The majority of biopsies were diagnostic and United Kingdom guidelines exist to minimize the volume of breast tissue removed in benign cases (Quality Assurance Guidelines for Surgeons in Breast Cancer Screening). Initial biopsy specimens were therefore small, the median weight of our recorded initial biopsies was only 20.5 g. To preserve cosmesis, cavity shavings were smaller than the main specimen in the majority of cases. In multifocal lesions at least, the surgical margin and or cavity shavings may lie between the tumour foci, giving the false impression of a free margin. Increasing experience with stereotactic FNAC will allow a preoperative diagnosis of malignancy to be made, and enable us to perform a therapeutic biopsy with a wider margin of excision. A recent study has explored the three-dimensional structure of the various types of DCIS using a stereoscopic technique (Faverly et al, 1994). This study showed that poorly differentiated DCIS nearly always grows continuously, although well-differentiated DCIS usually has a multifocal distribution. In cases of true multifocal disease, the gaps between foci are short (≤ 1 cm in 83% of cases). The authors of this study suggest that at the time of resection, a 1 cm rim of normal breast tissue should be excised around the primary lesion. With this approach, complete excision should be possible for approximately 90% of DCIS cases, irrespective of histological subtype.

Complete local excision is an essential requirement before entry into several on-going prospective trials of adjuvant therapy for DCIS, but no guidance as to the minimum margin of excision is given. Evidence from both this and previous studies suggest that this might lead to inadequate local excision and high local recurrence rates in some cases. We feel that to accurately assess the effect of adjuvant treatments such as tamoxifen or radiotherapy in preventing true DCIS relapse and progression, DCIS lesions must be completely excised. In this study, recurrence occurred in five patients receiving adjuvant tamoxifen therapy, however in the presence of almost certain residual disease, the efficacy of tamoxifen as a chemopreventative agent cannot be assessed.

Most authors now agree that recurrences after local excision of DCIS most likely reflect residual disease, and several studies clearly suggest that 1 mm is an inadequate margin of excision (Lagios et al, 1982; Holland et al, 1990; Silverstein et al, 1994; 1996). A tissue margin of 1–2 cm from the edge of the mammographically assessed lesion seems necessary to ensure that histological assessment of the margin is accurate. In our hands, biopsy cavity shavings have proved to be inadequate in assessing complete excision of screen-detected DCIS lesions using a clear margin of excision of 1 mm. With the aid of stereotactic FNAC, our clinical practice has now changed. We aim to perform a therapeutic biopsy at the initial procedure whenever possible, and proceed to elective wider excision if DCIS is close to the main specimen margin. We try to ensure a clear histological margin of 5–10 mm in each case; with such a margin, cavity shavings become irrelevant.

### REFERENCES

Faverly DRG, Burgers L, Bult P and Holland R (1994) Three dimensional imaging of mammary ductal carcinoma in situ: Clinical implications. *Sem Diagnos Pathol* **11**(3): 193–198

Fentiman IS, Fagg N, Millis RR and Hayward JL (1986) In situ ductal carcinoma of the breast: Implications of disease pattern and treatment. *Eur J Surg Oncol* **12**: 261–266

Fisher ER, Sass R, Fisher B, Gregorio R, Brown R and Wickerman L (1986) Pathologic findings from the National Surgical Adjuvant Breast Project (protocol 6). II. Relation of local breast recurrence to multicentricity. *Cancer* **57**: 1717–1724

Fisher ER, Leeming R, Anderson S, Redmond C and Fisher B (1991) Conservative management of intraductal carcinoma (DCIS) of the breast. *J Surg Oncol* **47**: 139–147

Fisher ER, Costantino J, Fisher B, Palekar AS, Redmond C and Mamounas E (1995) Pathologic Findings from the National Surgical Adjuvant Breast Project (NSABP) Protocol B-17. *Cancer* **75**: 1310–1319

Gump FE, Jicha DL and Ozzello L (1987) Ductal carcinoma in situ (DCIS): A revised concept. *Surgery* **102**: 790–795

Holland R, Hendricks JHCL, Verbeek ALM, Mravunac M and Schuurmans Stekhoven (1990) Extent, distribution and mammographic/histologic correlations of breast ductal carcinoma in situ. *Lancet* **335**: 519–522

Jenkins PL, May VE and Hughes LE (1991) Psychological morbidity associated with local recurrence of breast cancer. *Int J Psychiatry Med* **21**: 149–155

Kurtz JM, Jacquemier J, Amalric R, Brandone H, Ayme Y, Hans D, Bressac C, Roth J and Spitalier JM (1990). Risk factors for breast recurrence in premenopausal and postmenopausal patients with ductal cancers treated by conservation therapy. *Cancer* **65**: 1867–1878

Lagios MD, Westdahl PR, Margolin FR and Rose MR (1982) Duct carcinoma in situ: relationship of extent of noninvasive disease to the frequency of occult invasion, multicentricity, lymph node metastases, and short term treatment failures. *Cancer* **50**: 1309–1314

Macmillan RD, Purushotham AD, Mallon E, Ramsay G and George WD (1994) Breast-conserving surgery and tumour bed positivity in patients with breast cancer. *Br J Surg* **81**: 56–58

Price P, Sinnet HD, Gusterson B, Walsh G, A'Hern RP and McKinna JA (1989) Duct carcinoma in situ: can we predict recurrence after surgery? *Lancet* **ii**: 671–672

Price P, Sinnet HD, Gusterson B, Walsh G, A'Hern RP and McKinna JA (1990) Duct carcinoma in situ; predictors of local recurrence and progression in patients treated by surgery alone. *Br J Cancer* **61**: 869–872

Quality Assurance Guidelines For Surgeons In Breast Cancer Screening (1996) NHS Breast Screening Programme Publication number **20**

Silverstein MJ, Cohlan BF, Gierson ED, Furmanski M, Gamagami P, Colburn WJ, Lewinsky BS and Waisman JR (1992) Duct carcinoma in situ: 227 cases without microinvasion. *Eur J Cancer* **28A**: 630–634

Silverstein MJ, Gierson ED, Colburn WJ, Morin Cope L, Furmanski M, Senofsky GM, Gamagami P and Waisman JR (1994) Can intraductal breast carcinoma be excised completely by local excision? Clinical and pathological predictors. *Cancer* **73**: 2985–2989

Silverstein MJ, Lagios MD, Craig PH, Waisman JR, Lewinsky BS, Colburn WJ and Poller DN (1996). A prognostic index for ductal carcinoma in situ of the breast. *Cancer* **77**: 2267–2274

Verbeek ALM, Hendricks JHCL, Holland R, Mravunac M, Stummans F and Day NE (1984) Reduction of breast cancer mortality through mass screening with modern mammography: first results of the Nijmegen project. 1975–1981. *Lancet* **i**: 1222–1224

Veronesi U, Volterrani F, Luini A, Sacozzi R, Del Vecchio M and Zucali R (1990) Quadrantectomy versus lumpectomy for small size breast cancer. *Eur J Cancer* **26**: 671–673

Walls J, Knox F, Baildam AD, Asbury DL, Mansel RE and Bundred NJ (1995) Can preoperative factors predict for residual malignancy after breast biopsy for invasive cancer? *Ann R Coll Surg Engl* **77**: 248–251

Wazer D, Dipetrillo T, Schmidt-Ullrich R, Weld L, Smith TJ, Marchant DJ and Robert NJ (1992) Factors influencing cosmetic outcome and complication risk after conservation surgery and radiotherapy for early stage breast carcinoma. *J Clin Oncol* **10**: 356–363

© Cancer Research Campaign 1998